# Complete Genome Sequence of Pseudomonas aeruginosa Bacteriophage PASA16, Used in Multiple Phage Therapy Treatments Globally

**DOI:** 10.1128/mra.00092-22

**Published:** 2022-03-08

**Authors:** Sivan Alkalay-Oren, Ortal Yerushalmy, Karen Adler, Leron Khalifa, Daniel Gelman, Shunit Coppenhagen-Glazer, Ran Nir-Paz, Ronen Hazan

**Affiliations:** a Institute of Biomedical and Oral Research (IBOR), Faculty of Dental Medicine, Hebrew University of Jerusalem, Israel; b Department of Clinical Microbiology and Infectious Diseases, Hadassah-Hebrew University Medical Center Jerusalem, Israel; Queens College CUNY

## Abstract

PASA16 is a Pseudomonas aeruginosa phage isolated from a soil sample and used to treat several patients suffering from persistent infections in various countries. PASA16’s genome was sequenced, analyzed, and deposited in GenBank.

## ANNOUNCEMENT

Pseudomonas aeruginosa is an opportunistic pathogen which commonly causes infections in health care settings (1–3). Its antibiotic-resistant strains are among the most challenging to treat and reportedly cause 32,600 infections yearly and 2,700 deaths in hospitalized U.S. patients (4).

Phage therapy is currently one of the most promising solutions to the emergence of antibiotic-resistant bacteria in general and against P. aeruginosa in particular.

**FIG 1:**
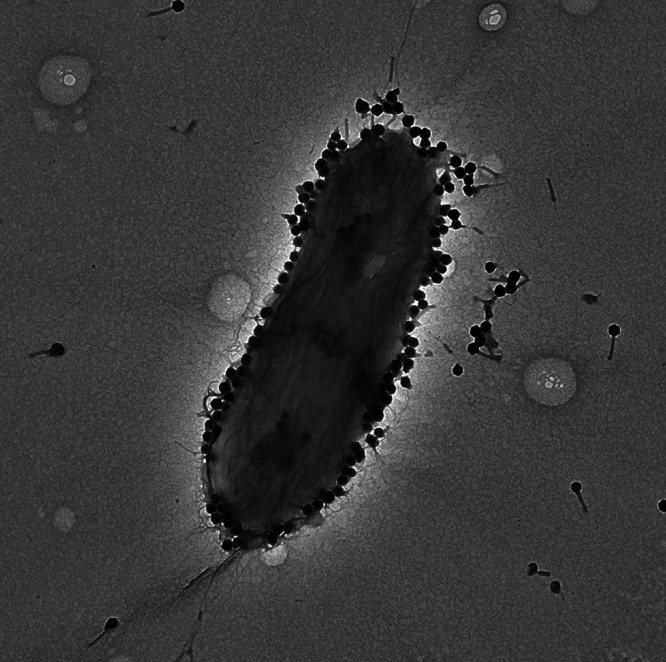
A TEM visualization of the phage PASA16 with P. aeruginosa PA14.

Here, we report the genome sequence of the anti-P. aeruginosa phage PASA16, used in several phage-therapy treatments (5). PASA16 was isolated in Jerusalem from a garden (31°45′42″N, 35°10′19″E) soil sample and purified using the phage titration method (6, 7). Briefly, the sample was dissolved in LB and mixed with overnight-grown P. aeruginosa PA14 at 37°C. The culture was centrifuged (7,100 × *g*, 10 min), and the supernatant was filtered (0.22 μm). Serial dilutions of 5 μL of the filtrate were spotted on P. aeruginosa PA14 lawn, and after overnight incubation at 37°C, the clearest plaques were picked, and their titer was determined. The phage particles were visualized by transmission electron microscopy (TEM) (Fig. 1) as described (6).

DNA was purified (phage DNA isolation kit; Norgen Biotek) from a single plaque that was picked and grown to a titer of 10^9^ PFU/mL, followed by library preparation (Illumina Nextera XT kit) and sequencing (Illumina NextSeq 500/550 kit) with single-end reads of 150 bp (Illumina 150-cycle midoutput kit v2,) according to Illumina’s recommendations. Machine output files (binary base call [BCL]) were converted to FASTQ files using the bcl2fastq/2.20.0 program.

Most bioinformatic analyses were performed using Geneious Prime v2021.0.3 and its plugins (Biomatters). The reads were trimmed (Geneious Prime BBDuk plugin) with default parameters. Sequencing of PASA16 yielded 1,651,174 reads of 150 bp in size. Bacterial sequences were removed by map-to-reference on the P. aeruginosa PA14 genome sequence followed by *de novo* assembly of the remaining reads with the SPAdes plugin of Geneious Prime with default parameters. The resulting contigs were reassembled with the Geneious Prime assembler with the highest sensitivity to produce one major contig of ∼60 kb, which was fine-tuned by 10 iterative cycles of map-to-reference of the trimmed reads and consensus generation. The average coverage was 3,580.1× with a standard deviation (SD) of 351.2.

The PASA16 genome is 66,127 bp long with a GC content of 55.6% and contains 90 coding DNA sequences (CDS). Its terminal repeats and analysis of the alignment of de- and reassembled genomes reveal that the PASA16 genome topology is either circular or circularly permuted (8). The taxonomy of PASA16, as determined by GenBank, is *Duplodnaviria*, *Heunggongvirae*, *Uroviricota*, *Caudoviricetes*, *Caudovirales*, *Myoviridae*, and *Pbunavirus.* Annotation with RAST (9) revealed that most PASA16 genes encode phage-related or hypothetical proteins. One notable gene is a lytic enzyme gene (GenBank protein accession number QOI69201.1) coding a putative lysozyme-like endolysin. This gene increases the possibility of the phage having high lytic abilities against the target bacteria (10) and perhaps contributed to its success in phage therapy treatments. As a candidate for phage therapy, the phage sequence was scanned for virulence factors using ABRicate v1.0.1 (T. Seemann, https://github.com/tseemann/abricate) against all its databases with its default parameters. No virulence factors or resistance genes were identified, and therefore the phage was deemed safe for use in phage therapy treatments.

### Data availability.

The sequence of the annotated genome of PASA16 can be found in GenBank (accession number MT933737.1), and the raw data are available under NIH BioSample database project PRJNA706131, “The Israeli Phage Bank (IPB),” BioSample number SAMN25300365, and Sequence Read Archive (SRA) number SRX13974252.
